# Targeted therapies in hepatocellular carcinoma: past, present, and future

**DOI:** 10.3389/fonc.2024.1432423

**Published:** 2024-08-29

**Authors:** Rushabh Gujarathi, Joseph W. Franses, Anjana Pillai, Chih-Yi Liao

**Affiliations:** ^1^ Section of Hematology and Oncology, Department of Medicine, University of Chicago, Chicago, IL, United States; ^2^ Division of Gastroenterology, Hepatology and Nutrition, Department of Internal Medicine, University of Chicago, Chicago, IL, United States

**Keywords:** hepatocellular carcinoma, targeted therapy, clinical trials, tyrosine kinase inhibitors, immunotherapy

## Abstract

Targeted therapies are the mainstay of systemic therapies for patients with advanced, unresectable, or metastatic hepatocellular carcinoma. Several therapeutic targets, such as c-Met, TGF-β, and FGFR, have been evaluated in the past, though results from these clinical studies failed to show clinical benefit. However, these remain important targets for the future with novel targeted agents and strategies. The Wnt/β-catenin signaling pathway, c-Myc oncogene, GPC3, PPT1 are exciting novel targets, among others, currently undergoing evaluation. Through this review, we aim to provide an overview of previously evaluated and potentially novel therapeutic targets and explore their continued relevance in ongoing and future studies for HCC.

## Introduction

Hepatocellular Carcinoma (HCC) is the most common type of primary liver cancer worldwide and accounts for over 75% of all cases of primary liver cancer (1). While the incidence of HCC and overall HCC-specific mortality have shown a slight decline in recent years, the prognosis remains poor, with an estimated 5-year relative survival rate of approximately 22% (2, 3). Analyses from the Global Burden of Diseases, Injuries, and Risk Factors Study (GBD) have identified notable changes in the underlying etiologies for HCC over the last three decades, with steady increases in the incidence and mortality rates of liver cancer linked to Hepatitis B viral infections (HBV), metabolic dysfunction-associated steatotic liver disease (MASLD), and alcohol use (2, 4).

Treatment options for HCC are greatly influenced by the patient’s disease stage, burden of disease, degree of liver dysfunction, and performance status. A multi-disciplinary approach allows for optimal patient selection for various treatment options and is widely considered the standard of care at most comprehensive cancer centers (5). Treatment modalities commonly used in clinical practice include surgical resection, liver transplantation, percutaneous ablation, transarterial therapies, external beam radiation, and systemic therapy (6). Patients with localized and early-stage disease (Barcelona Clinic Liver Cancer [BCLC] stage 0 or A) may be eligible for curative-intent treatment, including surgical resection, liver transplantation, or percutaneous ablation. Patients with unresectable and advanced/metastatic disease (BCLC stage B and C) can benefit from palliative systemic therapy (6). Certain patients with BCLC stage B who undergo treatment with effective downstaging strategies can potentially be eligible for liver transplantation (7). Systemic therapies for HCC are broadly subdivided into two major subgroups: targeted therapies and immune checkpoint inhibitors (ICIs). Targeted therapies are drugs that interfere with specific molecules or pathways that are involved in the growth, survival, and spread of cancer cells. Most targeted therapeutic agents are either monoclonal antibodies (mAbs) or drugs known as small molecule inhibitors (8). Sorafenib, a small-molecule multi-kinase inhibitor (mTKI), was the mainstay of systemic therapy in HCC for close to eleven years following its approval in 2007 (9, 10). Lenvatinib, another mTKI, was shown to be non-inferior and approved based on the results from the REFLECT trial (11) for use in advanced HCC in 2018. In 2020, results from the IMBrave150 study significantly changed the landscape of systemic therapy in HCC, and the combination of atezolizumab, an ICI that targets anti–programmed cell death ligand-1 (PD-L1), and bevacizumab, an anti-angiogenic mAb targeting vascular endothelial growth factor A (VEGF-A), has now been established as the first-line treatment for unresectable HCC worldwide (12). In 2022, the combination of durvalumab, an ICI targeting PD-L1, with tremelimumab, an ICI targeting cytotoxic T lymphocyte–associated antigen 4 (CTLA-4) was also approved as a first-line therapy option, based on the results of the HIMALAYA study (13). In the current HCC systemic therapy landscape, multiple targeted therapies are approved for use in the second-line and beyond setting, including but not limited to additional mTKIs (regorafenib and cabozantinib), the anti-angiogenic monoclonal antibody ramucirumab, and the combination of the ICIs nivolumab and ipilimumab (14–18).

The major differences between ICIs and targeted therapies lie in their adverse event profiles and mechanisms of action. Immune checkpoint inhibitors (ICIs) are drugs that disrupt the interaction between immune checkpoint proteins and their ligands, thus preserving the activation of T cells and their anti-tumor immunological activity (19). ICIs are said to be well-tolerated as compared to targeted therapies but can be associated with immune-related adverse events (irAEs) which are unpredictable and potentially long-term (20). The use of ICIs is discouraged in patients with moderate to severe autoimmune conditions, and the American Association for the Study of Liver Diseases (AASLD) advises against their use in post-transplant patients due to the high risk of graft loss and mortality (6).

Various other targeted therapeutic agents have been evaluated in HCC, though these studies have failed to establish clinical benefit. An intimate understanding of the agents that have been evaluated in the past, the reasons for their lack of meaningful efficacy, and the lessons learned from the respective trials is essential for developing more effective targeted therapy regimens in the future. In this review, we first provide a brief overview of the currently approved targeted therapies for HCC. Then, we summarize the major characteristics, trial data, and challenges relating to previously evaluated targeted therapeutic agents in HCC. Finally, we discuss notable ongoing clinical trials of novel targeted therapy approaches in HCC and the potential directions for future developments in the field.

## Sytemic therapy in HCC including approved targeted therapies – a brief overview

The preferred first-line systemic treatment option for patients with advanced, unresectable, or metastatic HCC involves immunotherapy combinations: atezolizumab and bevacizumab, or durvalumab and tremelimumab (6). The global, open-label, randomized phase III IMbrave 150 study established atezolizumab and bevacizumab as a preferred first-line treatment option in patients with advanced HCC, conferring a median overall survival (mOS) of 19.2 months (95% CI, 17.0 months – 23.7 months) with the combination, compared to 13.4 months (95% CI, 11.4 months – 16.9 months) with sorafenib (HR, 0.66; 95% CI, 0.52 – 0.85; p < 0.001) (12). The phase III HIMALAYA trial evaluated durvalumab and tremelimumab (given via the “STRIDE” dosing regimen, involving a single priming dose of tremelimumab with monthly durvalumab) versus sorafenib, and the STRIDE regimen led to a mOS of 16.4 months (95% CI, 14.2 months – 19.6 months) in the STRIDE arm, as compared to 13.8 months (95% CI, 12.2 months – 16.1 months) with sorafenib (HR, 0.78; 96.02% CI, 0.65 – 0.93; p = 0.0035) (13).

Among the currently approved mTKIs for use in HCC, sorafenib and lenvatinib are the only two agents recommended for use as first-line systemic therapy in HCC (6). Sorafenib inhibits tumor angiogenesis by targeting several kinases involved in oncogenic signaling, mainly RAF1, BRAF, the receptor tyrosine kinase activity of vascular endothelial growth factor receptors (VEGFRs) 1, 2, and 3, and platelet-derived growth factor receptor β (PDGFR-β) (21–23). Commonly reported adverse events with sorafenib therapy include hand–foot skin reaction, rash, diarrhea, hypertension, weight loss, and fatigue (9, 24, 25). Lenvatinib works in a similar fashion by inhibiting several kinase-mediated pathways involved in cancer cell proliferation and tumor angiogenesis. These include VEGFRs 1, 2, and 3, fibroblast growth factor (FGF) receptors 1, 2, 3, and 4, PDGF receptor α, RET, and KIT (26–28). Lenvatinib therapy shows adverse events similar to sorafenib, among which hand–foot skin reaction, rash, diarrhea, hypertension, weight loss, loss of appetite, and proteinuria are common (29). The incidence of hand-foot skin reaction and diarrhea was found to be higher with sorafenib therapy, and hypertension, decreased appetite, weight loss, fatigue, and proteinuria were found to be more commonly associated with lenvatinib therapy in a meta-analysis (25). The principal indication for sorafenib and lenvatinib in current practice is in patients with advanced HCC who are unable to receive the preferred first-line regimen(s) of atezolizumab and bevacizumab or durvalumab and tremelimumab. These patients usually have contraindications for immune checkpoint inhibitors or intolerance to these agents (6).

Several targeted therapy agents are approved in the second-line and beyond setting for patients with disease that has progressed on first-line treatment options. Regorafenib is an mTKI which mainly acts on VEGFRs 1, 2, and 3, TIE2, PDGFR, FGFRs 1 and 2, KIT, RAF1, BRAF, and RET (30). The phase III RESORCE study evaluated regorafenib in HCC patients who had developed disease progression on sorafenib treatment. Regorafenib use resulted in a mOS of 10.6 months (95% CI, 9.1 months – 12.1 months) which was superior to the mOS of 7.8 months (95%CI, 6.3 months – 8.8 months) with placebo (14). The most common clinically relevant grade 3 or 4 adverse events reported in this study with regorafenib were hypertension, hand-foot skin reaction, and diarrhea (14). Cabozantinib, an mTKI that inhibits several kinases – including MET, AXL, RET, FLT3, and VEGFR2 - was approved for use in patients with HCC who had been previously treated with sorafenib based on results from the phase III CELESTIAL trial. The mOS was 10.2 months (95% CI, 9.1 months – 12.0 months) in the cabozantinib group versus 8.0 months (95% CI, 6.8 months – 9.4 months) with HR 0.76 (95% CI, 0.63 – 0.92; p = 0.005). Progression-free survival (PFS; HR, 0.44; 95% CI, 0.36 – 0.52; p < 0.001) and objective response rate (ORR; 4% vs. <1%; p = 0.009) were also found to significantly favor cabozantinib over placebo in the study (15). The most common grade 3 or 4 adverse events reported in the cabozantinib group were hand-foot skin reaction (described as palmar–plantar erythrodysesthesia), hypertension, increased aspartate aminotransferase level, and diarrhea (15). The efficacy of cabozantinib as first line therapy in combination with atezolizumab was investigated in the multicenter, open-label, randomized, phase III trial COSMIC-312, but the difference in mOS with the combination (16.5 months; 96% CI, 14.5 months – 18.7 months) as compared to sorafenib monotherapy (15.5 months; 96% CI, 12.2 months – 20 months) was not statistically significant (HR, 0.98; 96% CI, 0.78 – 1.24; p = 0.87) (31).

Ramucirumab, a human monoclonal antibody against VEGFR2, was evaluated for efficacy and safety in the global, randomized, double-blind, placebo-controlled, phase III REACH study (16). Although no significant benefit in mOS was seen in the study population as a whole, the predefined subgroup of patients having baseline alpha fetoprotein (AFP) levels above 400 ng/mL showed a significant improvement in mOS (HR, 0.67; 95% CI, 0.51 – 0.90; p = 0.006). The subsequent randomized, double-blind, placebo-controlled, phase III REACH-2 study further validated these findings. In advanced HCC patients with baseline AFP levels above 400 ng/mL who had progressed on prior sorafenib therapy, mOS (HR, 0.71; 95% CI, 0.53 – 0.95; p = 0.0199) and PFS (HR, 0.45; 95% CI, 0.34 – 0.60; p < 0.0001) were found to significantly favor ramucirumab monotherapy over placebo (16, 17). In the ramucirumab group, hypertension and hyponatremia were the only grade 3 or worse treatment-emergent adverse events observed in 5% or more of patients and occurring at higher frequencies than in the placebo group (17).

It is worthwhile to note that the adverse events seen with the use of targeted therapies are different from the irAEs usually seen with the use of ICIs. These reactions are said to be unpredictable, potentially long-term and can affect any organ system (20). Commonly seen irAEs in HCC include fatigue, pyrexia, rash, pruritus, diarrhea, decreased appetite, nausea, abdominal pain, constipation, hepatitis, and hypothyroidism (20).

Notably, all the prospective trials mentioned above were restricted to patients with a good performance status (ECOG 0-1) and limited liver dysfunction (Child-Pugh, CP, class A). Clinical data for patients with CP class B disease are limited, but they highlight the unmet need to evaluate these and additional agents in patients with CP class B liver function in future trials (32, 33).

## Notable therapeutic targets: historical perspectives and future directions

### c-Met

The *MET* (MET proto-oncogene, receptor tyrosine kinase) gene is responsible for encoding the c-Met (mesenchymal-epithelial transition factor) protein, which is the cell surface receptor for hepatocyte growth factor (HGF). The expression of c-Met is seen on epithelial cells, endothelial cells, neurons, hepatocytes, and hematopoietic cells (34). A complex interplay exists between HCC and the cellular functions regulated by c-Met. Liver disease is known to increase demands for hepatocyte proliferation, which in turn promotes the up-regulation of c-Met activity. Although this increase in activity is beneficial in chronic liver disease, an aberrant increase in c-Met activity results in oncogenic cellular effects and contributes to tumorigenesis, proliferation, and metastasis in HCC (34–36). A significant increase in *MET* mRNA expression is seen in hepatitis, liver cirrhosis, and HCC (37). Although the c-Met protein was found to be overexpressed in 27.9% of HCC tumor specimens (from 287 patients with HCC), there was no prognostic impact of this finding in patients after surgical resection (38). c-Met/HGF and related pathways also play a role in the development of resistance to sorafenib therapy in patients with HCC (39–41). c-Met expression was hence thought to be an important therapeutic target in HCC and led to trials with agents inhibiting the cellular effects of c-Met activation by HGF in patients with HCC. While the currently-approved agent cabozantinib also inhibits c-Met activity as part of its spectrum of kinase inhibition, several clinical trials have assessed the utility of more potent c-Met inhibitors – including tivantinib, tepotinib, capmatinib, and golvatinib - have been completed (42).

Tivantinib (ARQ 197) is an oral, small-molecule inhibitor of c-Met that has been shown to inhibit intratumoral c-Met signaling in tumor biopsy samples and is thought to preferentially act upon cell lines expressing c-Met to induce apoptosis and exert anti-tumor effects (43, 44). The safety and efficacy of tivantinib monotherapy for patients with previously treated (with sorafenib) HCC having tumors with high levels of c-Met (≥ 2+ expression in ≥ 50% of tumor cells by IHC) was studied in two large randomized, double-blind, placebo-controlled studies: the METIV-HCC and JET-HCC studies. The METIV-HCC study was conducted across ninety centers in Australia, the Americas, Europe, and New Zealand, in which 340 patients with c-Met-high HCC were enrolled, and 226 patients received tivantinib. The JET-HCC study was conducted at sixty centers in Japan, where 194 patients were eventually randomized to receive either tivantinib (n = 133) or placebo (n = 61). Final analysis from the METIV-HCC study demonstrated a mOS of 8.4 months (95% CI, 6.8 months – 10.0 months) in the tivantinib group compared to 9.1 months (95% CI, 7.3 months – 10.4 months) with placebo, and the difference in risk of death was not significant (HR, 0.97; 95% CI, 0.75 – 1.25; p = 0·81) across a median follow-up period of 18.1 months (IQR, 14.1 months – 23.1 months) (45). Similar results were reported from the JET-HCC study, where mOS was 8.5 months (95% CI, 6.2 months – 11.4 months) in the tivantinib group and 10.3 months (95% CI, 8.1 months – 11.4 months) in the placebo group, with an insignificant difference in risk (HR, 0.82; 95% CI, 0.58 – 1.15) (46).

Tepotinib, an oral highly selective inhibitor of c‐Met/HGF signaling, was also assessed for safety and efficacy in two phase Ib/II studies for HCC patients. Tepotinib exerts its anti-tumor activities in a manner different to tivantinib. Tivantinib is a non-ATP competitive inhibitor of c-Met, while tepotinib competes with adenosine triphosphate (ATP) for binding to the receptor, thus preventing the phosphorylation and subsequent activation (47). A single-arm phase Ib/II study conducted across various centers in Europe and the US showed a 12-week PFS rate of 63.3% (versus historical pre-specified control of 15%, p < 0.0001) with tepotinib. Although none of the observed trends were found to be statistically significant, the investigators reported that a trend towards better PFS at 12 weeks in patients with c-Met IHC 3+ (versus 2+), *MET* amplification (versus no *MET* amplification), AFP elevation at baseline of more than 200 mcg/L, and Hepatitis B/C virus positivity was seen on subgroup analyses from the phase II cohort (48). Similarly, a phase Ib/II study of tepotinib conducted in China, South Korea, and Taiwan showed a significant improvement in independently assessed time to progression (TTP) in patients treated with tepotinib versus sorafenib (HR = 0.42, 90% CI, 0.26 – 0.70, p = 0.0043). Although the confidence interval reported was larger than the usually accepted 95%, such modifications are often seen in trials with similarly small sample sizes (49). A notable difference between the trials was that the western study described aimed to evaluate the efficacy of tepotinib in the second-line setting (after sorafenib), whereas the Asian study investigated its use as first-line systemic therapy. Although both studies met their primary endpoints in phase II and the results seemed promising, a phase III study was not undertaken for tepotinib due to the observed modest effect sizes and a limited patient pool.

Another highly specific competitive inhibitor of c-Met, capmatinib, was evaluated for safety and efficacy in HCC patients with tumors showing *MET* dysregulation who had not received prior systemic therapy. Notably, the criteria for c-Met positivity were modified midway through the study in light of preliminary data indicating that high c-Met protein expression and increased *MET* gene copy number predicted response to capmatinib. The phase II, open-label, single-arm, multicenter study was conducted across the Asia-Pacific region. Unfortunately, study enrollment was prematurely halted due to challenges in identifying eligible patients. The primary endpoint of time to progression was not analyzed due to a limited sample size (50).

A dual inhibitor of c-Met and VEGF, golvatinib, exhibited strong inhibition of tumor growth and angiogenesis in xenograft models (51). The combination of golvatinib with sorafenib was evaluated in comparison to sorafenib monotherapy for patients with advanced or metastatic HCC in an open-label, randomized phase Ib/II study that completed accrual in 2015 (NCT01271504). Phase I results reported partial response (PR) in two patients and stable disease in three patients out of 13 patients enrolled at two dose levels (52). Further studies for golvatinib in HCC have not been undertaken.

Emibetuzumab, an anti-c-Met bivalent antibody that works by inhibiting the ligand-dependent and ligand-independent initiation of c-Met/HGF signaling, was evaluated for safety and efficacy in combination with ramucirumab in several solid tumors in a two-part, multicenter, nonrandomized, open-label phase Ib/II study (53). The study included 45 patients with HCC, of whom 37 had received prior sorafenib. Within the HCC cohort, a median PFS (mPFS) of 5.4 months (95% CI, 1.6 months – 8.1 months) was reported. Subgroup analysis of HCC patients with high c-Met expression showed that HCC patients with tumors having c-Met expression of 2+ or more in at least 50% of tumor cells had a mPFS of 8.1 months compared to patients below this expression cut-off, who had a mPFS of 2.8 months. The risk of progression was significantly reduced in the c-Met-high group as well (HR, 0.22; 90% CI, 0.08 – 0.59) (54). While further studies with emibetuzumab have not been undertaken, potentially due to a lack of meaningful anti-tumor activity and/or patient selection difficulties, these results highlight the potential utility of biomarker-selected trials for HCC.

While some of these studies showed promise in their respective settings, an overall lack of meaningful clinical efficacy with selective c-Met inhibition (tepotinib, capmatinib) or drugs with more promiscuous activity that includes c-Met inhibition (tivantinib, golvatinib) may be attributed to several factors. Difficulty in accrual and modest effect sizes seem to be the most likely reasons for the discontinuation of most investigations. However, results from these studies demonstrate an overall trend towards improved outcomes within biomarker-selected populations, highlighting the importance of biomarker-driven patient selection for future trials. Hence, the findings from these studies should not be interpreted as evidence of the ineffectiveness of c-Met inhibition in HCC, but rather should be used to guide future study design.

c-Met inhibition remains an important therapeutic target in HCC as indicated by several ongoing studies. Promising novel c-Met-targeted approaches include chimeric antigen receptor T-cell (CAR-T) therapy and Antibody-drug conjugates (ADCs). Jiang et al. reported the successful construction of dual-targeting c-Met/PD-L1 (CP) CAR-T cells and found that the bispecific CAR-T cells had promising anti-tumor effects in HCC cells, and these effects were suggested to be more potent than those of monovalent c-Met targeting CAR-T cells (55). ADCs exploit the targeted delivery of a drug, usually a cytotoxic payload, to cancer cells expressing an antigen to which the antibody portion of the ADC binds selectively. The two are connected by a linker molecule, and the antibody-mediated binding results in internalization of the ADC, causing targeted drug release (56). Targeting the c-Met pathway via ADCs has shown promise in preclinical studies. ABBV-400, an ADC composed of the c-Met–targeting antibody telisotuzumab conjugated to a potent proprietary topoisomerase 1 inhibitor (Top1i) payload, showed promising efficacy in several solid tumor models (57). In the dose-escalation study involving 47 patients with various solid tumors receiving ABBV-400, the ORR was 24.4% (95% CI, 12.9% - 39.5%) (58). Further clinical investigation of this ADC in HCC has been initiated. In addition to directly inhibiting the c-Met/HGF pathway, employing c-Met as a target for Antibody-Drug Conjugates (ADCs) to deliver chemotherapy drugs such as oxaliplatin and doxorubicin has demonstrated activity in preclinical HCC models (59, 60). Finally, the combination of c-Met inhibition with immune checkpoint inhibitors warrants further clinical investigation. Preclinical studies have demonstrated that combining anti-PD1 with c-Met inhibition (capmatinib or tivantinib) may lead to promising outcomes, decreasing tumor growth and prolonging survival in mice with orthotropic tumors, compared with anti-PD1 or c-Met inhibitors alone. c-Met inhibition may also help potentially overcome immune checkpoint blockade resistance in HCC, paving the way for future clinical studies involving anti-PD1 with c-Met inhibition (61, 62).

### TGF-β

Transforming growth factor-β (TGF-β) is a cytokine that is involved in the regulation of numerous cellular processes, including but not limited to apoptosis, angiogenesis, cellular differentiation, inflammation, and proliferation. Signaling pathways linked to TGF-β are also known to regulate the maintenance of genomic stability and stem cell homeostasis (63, 64). The TGF-β signaling pathway involves SMAD proteins as key intracellular effectors and SMAD-dependent downstream signaling pathways constitute the canonical cellular signaling pathways regulated by TGF-β. Non-canonical signaling pathways, including the MAPK pathways such as ERK, JNK, and p38 MAPK, can also be activated by TGF-β (65, 66). TGF-β signaling also exhibits duality of function, which renders it challenging to target TGF-β through anti-cancer therapies. TGF-β signaling pathways act as tumor suppressors in normal cells and early carcinomas by controlling cell growth, death, and immortalization (67). As tumors evolve, the protective and cytostatic effects of TGFβ are frequently diminished. Subsequently, TGF-β signaling promotes angiogenesis, tumor progression, invasion, and metastasis (67, 68). Additionally, TGF-β is often referred to as the “master regulator” for immune cell proliferation, differentiation, development, and survival (63, 64, 66, 69). TGF-β promotes the production of proinflammatory Th17 cells, which contribute to MASLD-associated inflammation and hepatocarcinogenesis (70, 71). TGF-β also acts as an inhibitor for type I helper T (Th1) lymphocytes and type II helper T cell (Th2) lineages, leads to a reduction in interferon gamma expression, and suppresses natural killer (NK) T cells (72–74). During B cell maturation, TGF-β and runt-related transcription factor 3 (RUNX3) facilitate the class switching of naïve B cells to immunoglobulin A (IgA) producing cells, which is implicated in MASLD-associated HCC, through PD-L1 and IL-10 coexpression and the suppression of hepatic cytotoxic CD8+ T lymphocytes (75). Furthermore, TGF-β facilitates the differentiation of M2-type macrophages, which leads to an increase in the activity of CD4+ regulatory T lymphocytes (Tregs) and the suppression of CD8+ T cell and NK cell activity, along with reduced antigen presentation on dendritic cells (64, 76). The role of chronic inflammation in hepatocarcinogenesis, often associated with liver fibrosis and cirrhosis, is well established (74). TGF-β signaling also plays a role in the activation of cancer-associated fibroblasts, which further highlights its role in hepatocarcinogenesis (77, 78). Tumor cell secreted TGF-β also upregulates the expression of programmed cell death protein 1 (PD-1), thus playing a role in the induction of tumor cell immunosuppressive mechanisms, particularly in virally induced cancers (79). TGF-β regulates the epithelial mesenchymal transition (EMT), angiogenesis by modulating VEGF, and tumor cell vascular invasion by activating β1 integrin, among other cellular processes, and promotes tumor progression in HCC (80–82). Within the liver, TGF-β signaling mediates various stages of disease progression, including initial liver injury, inflammation, fibrosis, cirrhosis, and finally cancer. TGF-β is thought to behave as a tumor suppressor in the early stages of liver tumorigenesis, but evidence suggests that TGF-β signaling can contribute to tumor progression later, once cells are able to overcome its cytostatic effects (83). TGF-β levels are often found to be elevated in serum samples from patients with HCC (84). An increase in growth factor sensitivity modulated by TGF-β is also thought to contribute to sorafenib resistance in patients with HCC (85).

Perhaps the most widely studied drug in this class for HCC was the oral, small-molecule selective inhibitor of the TGF-β receptor type I (RI), galunisertib. The utility of galunisertib has been studied mainly as first-line combination therapy with sorafenib in advanced HCC, in combination with stereotactic body radiation therapy (SBRT) for advanced HCC, and briefly in combination with the ICI nivolumab for advanced disease. A phase II study evaluated galunisertib in four cohorts. In part A, 109 patients with AFP elevations >1.5 times the upper limit of normal received galunisertib (randomized to either 80 mg or 150 mg, twice per day). Part B included 40 patients with lower AFP levels who received 150 mg twice per day. The part B group had a longer TTP (4.2 months; 95% CI, 1.7 months – 5.5 months) compared to part A (2.7 months; 95% CI, 1.5 months – 2.9 months) (86). Part C explored galunisertib combined with sorafenib in patients with advanced HCC, and the reported safety profile was comparable to sorafenib monotherapy. Among 44 patients receiving 150 mg galunisertib, the mTTP was 4.1 months (95% CI, 2.8 months – 6.5 months), with increased TTP associated with declining TGF-β1 levels on treatment (87). The combination galusertinib plus nivolumab was being evaluated in non-small cell lung cancer (NSCLC) and HCC in a phase Ib/II study that reported preliminary efficacy in a subset of NSCLC patients. However, the HCC cohort of the study was terminated prematurely due to insufficient enrollment (88). Reiss et al. reported a favorable safety profile with the combination of galunisertib with SBRT for advanced HCC in a single-center pilot study. The only grade 3 adverse event reported was achalasia in one patient out of 15. Two instances of grade 2 alkaline phosphatase increase and two instances of grade 2 hyperbilirubinemia were reported (89). Galunisertib clinical development was discontinued in 2020 (90).

SAR439459 (SAR459), a monoclonal antibody against all isoforms of TGF-β, was investigated for use as both monotherapy and in combination with the ICI cemiplimab in patients with advanced solid tumors. The results from 14 patients with HCC who received the combination were reported, and only one patient showed an objective response. Progressive disease was the best response in six patients from the HCC cohort. Along with unsatisfactory tumor activity, there was a high reported bleeding risk, which was particularly pronounced in patients with HCC, where 11 out of the 14 patients reported a hemorrhagic adverse event. Further investigations were not undertaken in light of these findings (91). Bintrafusp alfa is a first‐in‐class bifunctional fusion protein composed of the extracellular domain of the TGF‐β RII receptor fused to a monoclonal antibody blocking programmed death‐ligand 1 (PD‐L1). An open label phase I dose‐escalation and expansion trial of bintrafusp alfa in Asian patients with metastatic or locally advanced solid tumors was conducted, which included nine patients with HCC. One patient was reported to have stable disease in the HCC cohort, and the safety profile was consistent with expected safety outcomes. Grade 3 or worse treatment related adverse events (TRAEs) were reported in three patients (grade 4 hyponatremia and grade 3 hypopituitarism; grade 3 intracranial tumor hemorrhage; and grade 3 increased blood creatine phosphokinase level, hyponatremia, and hypoacusis) in the evaluable cohort of 23 patients. No treatment‐related deaths or TGF‐β–related skin adverse events were reported in the HCC cohort. The activity of the drug was found to be insufficient by its manufacturer in three trials, and further investigations were discontinued (92, 93).

The TGF-β pathway remains an avenue of potential promise. As the first-line regimen for advanced HCC now includes ICI use for most patients, the utility of targeting the “master regulator” of immune responses may be effective in this setting. The combinations of future agents targeting TGF-β with ICIs may potentially represent adjuncts or modifications to existing first-line therapies, and trials designed in this setting may also overcome the issues with accrual seen in previous studies. Preclinical data has shown that the simultaneous blockade of TGF-β signaling with VEGF blockade can reinvigorate the anti-tumor immune response with ICIs. A novel combined anti-TGF-β/VEGF bispecific antibody, Y332D, in addition to PD-1 blockade, exhibited potent and durable anticancer effects in a variety of cancer cell lines. Clinically, this may translate to dual blockade helping overcome resistance to ICIs in these patients (94).

GARP (glycoprotein-A repetitions predominant) is a transmembrane cell surface receptor for TGF-β that is abundantly expressed on regulatory T lymphocytes and platelets. It is known to be a critical regulator of the activation of latent TGF-β (95). The oncogenic effects of the GARP and TGF-β axis have been shown in breast cancer orthotopic models. The selective blockade of TGF-β1 production by regulatory T-cells achieved by antibodies against GARP: TGF-β1 complexes was also reported to cause tumor regression in mouse models with tumors otherwise resistant to anti-PD-1 immunotherapy (95–97). Livmoniplimab (ABBV-151) is a first-in-class mAb targeting the GARP: TGF-β1 complex that leads to the blockade of the release of active TGF-β1. Results from a phase I study (NCT03821935) of livmoniplimab in combination with budigalimab, an anti-PD-1 mAb, showed an adverse event profile consistent with expected safety outcomes (98). The combination is being investigated for use as second-line therapy in advanced or metastatic HCC patients who have progressed on an ICI-containing first-line regimen in a phase II randomized study (NCT05822752), where patients will be randomized to two different dosing regimens of livmoniplimab with budigalimab, compared to a control arm of lenvatinib or sorafenib monotherapy. This study represents an extremely relevant clinical scenario in the current treatment landscape of HCC, where an effective first-line regimen is in use and patients progressing on the current first-line regimen require effective therapies (99). Similarly, the ongoing LIVIGNO-2 study (NCT06109272) is evaluating the optimum dosing, safety, and efficacy of the combination of livmoniplimab with budigalimab as a first-line regimen for locally advanced or metastatic HCC.

### FGFR

The fibroblast growth factor (FGF) pathway plays a regulatory role in several cellular processes that affect cell growth, survival, differentiation, and migration (100). The fibroblast growth factor receptor (FGFR) family of tyrosine kinases consists of four transmembrane proteins, FGFR1-4. Twenty-two known FGFR ligands (FGFs) exist, yet only 18 have been shown to induce the dimerization of these receptors and activate cell signaling pathways downstream. There is a fifth receptor (FGFR5) that lacks a tyrosine-kinase domain and is postulated to be a co-receptor of FGFR1 that affects its activity in response to ligands (100–102). The effectors of FGFR lead to the downstream activation of four intracellular pathways: mitogen-activated protein kinase (MAPK); phosphatidylinositol 3-kinase (PI3-kinase), phospholipase Cγ (PLCγ), and signal transducer and activator of transcription (STAT). The activation of these pathways plays an important role in cancer cell proliferation, angiogenesis, and metastasis in HCC (103). Increased pre-operative FGF2 levels are associated with tumor invasiveness and early post-operative disease recurrence in patients with HCC undergoing resection (104).

Brivanib, a selective dual inhibitor of the VEGF and FGF receptor families, has demonstrated antiangiogenic and antiproliferative effects in HCC xenograft models (105). The utility of brivanib as first-line therapy in treatment-naïve advanced HCC was first evaluated in the phase III BRISK-FL study. Brivanib was reported to be less well-tolerated than sorafenib, and the study did not meet its primary endpoint of non-inferiority to sorafenib (106). The phase III BRISK-PS study investigated the utility of brivanib as second-line therapy for patients who received prior sorafenib and had disease progression or intolerance to treatment. Although benefits were seen in the secondary endpoints of TTP (HR, 0.56; 95% CI, 0.42 – 0.76; p < 0.001) and objective response rate (10% vs 2%; p = 0.003), the primary endpoint of OS showed no improvement with brivanib over placebo (107). Despite these results, the inhibition of FGFR remains a potentially important therapeutic target for HCC. Lenvatinib and regorafenib both exert their anti-tumor effects partly through the inhibition of the FGFR signaling cascade (108, 109).

It is notable that the inhibition of individual FGFR receptors, from FGFR1 to FGFR4, is thought to have different therapeutic implications. FGFR3 and FGFR4 are the major FGFR isoforms overexpressed in HCC and represent potential pharmacological approaches (110–112). Zhao et al. reported the results of a study utilizing FGF19-positive HCC relevant xenograft and patient-derived xenograft models, which showed that the combined use of lenvatinib plus VEGFR2 antibodies with H3B-6527, a highly selective covalent FGFR4 inhibitor, strongly enhanced the efficacy of the selective FGFR4 inhibitor (113). Roblitinib (FGF401), a reversible and highly selective inhibitor of FGFR4, was evaluated for efficacy alone and in combination with spartalizumab, an anti-PD-1 antibody, in a phase I/II study (NCT02325739). 2 out of 12 patients who received the combination of roblitinib with spartalizumab showed a partial response (114). Although the study was halted due to commercial reasons, the utility of selective FGFR4 inhibitors is an ongoing subject of investigation. Fisogatinib (BLU-554), a highly selective oral irreversible FGFR4 inhibitor, showed clinical activity in a phase I study exclusively in HCC patients with FGF19 staining positivity by IHC (115). H3B-6527 was evaluated for safety in a phase I trial (NCT02834780), and interim analyses for HCC patients who had received two prior lines of treatment reported a mPFS of 4.1 months and a clinical benefit rate (defined as proportion of patients with objective response or patients with stable disease for a minimum of 17 weeks) of 45.8% (116).

These results, along with the results of the previously mentioned trials involving selective FGFR4 inhibitors, indicate that FGFR4 inhibition represents a potentially effective treatment option, potentially in combination with VEGF inhibition, and highlight the benefits of biomarker-based therapeutic selection. The evaluation of futibatinib, a highly selective irreversible inhibitor of FGFR1-4 approved for use in intrahepatic cholangiocarcinoma, in combination with pembrolizumab, is ongoing (NCT04828486) in HCC patients with tumors with demonstrated FGF19 expression (117).

### EGFR (with VEGF)

The epidermal growth factor receptor (EGFR) signaling pathway is frequently altered in HCC and plays an important role in tumorigenesis. EGFR links various inflammatory pathways involving liver injury with hepatocarcinogenesis. Elevated levels of ADAM17, which is an enzyme catalyzing the extracellular domains of EGFR, have been reported in models of liver injury prior to carcinogenesis (118, 119). Similarly, VEGF plays a critical role in tumor angiogenesis in HCC, and elevated levels of serum VEGF have been described as a biologic marker of tumor invasiveness and prognosis in HCC (120, 121). The combined effects of EGFR inhibition with VEGF using the combination of erlotinib, a potent selective EGFR/HER-1-related tyrosine kinase enzyme, and bevacizumab, a VEGF-inhibiting antibody, have shown promising results (122). A meta-analysis, which included eight phase II studies and a total of 342 HCC patients, showed a 16-week PFS rate of 50.2% (95% CI, 38.2% - 62.2%), and a 12-month OS rate of 44.9% (95% CI, 36.8% - 53.0%) (123). Although the combination is not currently used in clinical practice, these results indicate a potential utility for the combination, especially in patients who are ineligible for ICIs but in whom the beneficial effects of potent VEGF inhibition may prove useful. EGFR inhibition was also shown to reverse resistance to lenvatinib *in-vitro* in reports by He et al. (124) and Jin et al. (125). Furthermore, in a subsequently conducted clinical study that included twelve patients who progressed on initial lenvatinib therapy, subsequent treatment with a combination of lenvatinib and gefitinib yielded a partial response in four patients and stable disease in another four (125). The combination may represent untapped clinical potential, especially for patients who are ineligible to receive the current ICI + VEGF inhibitor combination in whom lenvatinib plays a major therapeutic role.

A summary of notable studies for previously evaluated targeted therapy is presented in **Table 1**. **Table 2** provides an overview of future directions with previously evaluated targets.

**Table 1:** Notable studies and results for previously evaluated molecular targets.

Target	Drug Name	Study Phase	HCC patients	Start/End of Enrollment	Locations	Brief Study Description	Notable results for Objective Response/Disease Control	Notable results for Progression-free survival	Notable results for Overall Survival	Limitations (as noted by authors and upon review)	Ref(s)
*MET*/c-Met	Tivantinib	III	340	Start: 12/2012 End: 12/2015	Australia, the Americas, Europe, and New Zealand	Tivantinib vs. Placebo in previously treated HCC with high c-Met expression	ORR = 0; DCR = 50%	HR = 0.96 (95% CI, 0.75–1.22; p = 0.72)	HR = 0.97 (0.75–1.25; p = 0.81)	Tivantinib formulation changed after phase 2.	(45)
	Tivantinib	III	195	Start: 1/2014 End: 6/2016	Japan	Tivantinib vs. Placebo in previously treated HCC with high c-Met expression	ORR = 0.7%; DCR = 67.2%;	HR = 0.74 (95% CI, 0.52 – 1.04; p = 0.082)	HR = 0.82 (0.58 – 1.15)	Selection bias due to biomarker test required for inclusion	(46)
	Tepotinib	Ib/II	49 (in II)	Start: 5/2014 End: 2/2017	Europe, USA	Tepotinib in previously treated HCC with c-Met overexpression	ORR = 10.2%; DCR = 49%	12-week PFS = 63.3% (90% CI, 50.5 – 74.7)	NA	Small number of patients with MET IHC 3+ staining or MET amplification	(48)
	Tepotinib	Ib/II	90 (in II)	Start: 2/2014 End: 08/2017	China, South Korea and Taiwan	Tepotinib vs. Sorafenib in treatment-naïve HCC with c-Met overexpression	ORR = 10.5%; DCR = 50%	HR = 0.42 (90% CI, 0.26 – 0.70, p = 0.0043)	NA	Small number of patients with MET IHC 3+ status. Underpowered due to early termination of enrollment	(49)
	Capmatinib	III	30 (in dose-expansion)	Start: 3/2013 End: 2/2017	China, Singapore, Thailand	Capmatinib in systemic treatment-naïve HCC with *MET* dysregulation, not suitable for or progression following locoregional therapy	ORR = 10%; DCR = 33%	Primary endpoint (TTP) not met;	NA	Enrollment halted after revision of criteria for MET positivity	(50)
	Golvatinib	I	13	Start: 07/2011 End: 06/2015 (from clinicaltrials.gov)	Europe, USA	Golvatinib in previously treated HCC	ORR = 16.67%	NA	NA	Limited sample size Phase II results not published	(52)
	Emibetuzumab	Ib/II	45 (in II)	Start: 03/2014 End: 12/2017 (from clinicaltrials.gov)	USA	Emibetuzumab plus Ramucirumab in advanced solid tumors	ORR (for HCC) = 7%; DCR (for HCC) = 60%;	HCC: 9-month PFS = 32.6% (95% CI, 16.7 – 49.5)	NA	Single arm design with multiple solid tumor types and no biomarker-based selection	(54)
TGF-β	Galunisertib (Parts A and B)	II	147	Start: 03/2011 End: 06/2019 (from clinicaltrials.gov)	Europe, USA, Australia, New Zealand	A: Galunisertib in previously treated HCC with AFP ≥ 1.5xULN B: Galunisertib in previously treated HCC with AFP <1.5xULN	NA	Part A 150mg vs. Part B 150mg: HR = 1.5 (95% CI, 1.0 – 2.2)	Part A 150mg vs. Part B 150mg: HR = 2.1 (95% CI, 1.3 – 3.3)	Single arm design and comparison with historical control only	(86)
	Galunisertib (Part C)	II	44	Start: 03/2011 End: 06/2019 (from clinicaltrials.gov)	Europe, USA, Australia, New Zealand	C: Galunisertib and Sorafenib in systemic treatment-naïve HCC	ORR = 4.5%; DCR = 52.2%	mTTP = 4.1 months (95% CI, 2.8 – 6.5)	mOS = 18.8 months (95% CI, 14.8 – 24.8)	Single arm design and no evaluation of peripheral regulatory T-cell counts	(87)
	SAR439459	I/Ib	14	Start: 10/2019 End: 09/2021	Europe, USA, Canada, Australia, Asia	SAR439459 plus Cemiplimab in solid tumors including HCC	ORR (for HCC) = 4.2%; DCR (for HCC = 25%)	NA	NA	Study discontinued due to lack of efficacy and bleeding risk	(91)
	Bintrafusp alfa	I	9	Start: 03/2016 End: 08/2018	Asia	Bintrafusp alfa in Asian patients with advanced solid tumors	ORR (for HCC) = 0; DCR (for HCC) = 11.1%)	NA	NA	Small number of HCC cases	(92)
FGFR	Brivanib	III	1150	Start: 05/2009 End: 08/2011	Australia, the Americas, Asia, Europe, and Africa	Brivanib vs. Sorafenib in systemic treatment-naïve advanced HCC	ORR = 12%; DCR = 66%;	HR = 1.01 (95% CI, 0.88 – 1.16; p = 0.85)	HR = 1.07 (95%CI, 0.94 – 1.23; p = 0.31)	No biomarker-based selection (as older study)	(106)
	Brivanib	III	395	Start: 02/2009 End: 06/2011	Asia, the Americas, and Europe	Brivanib vs. Placebo in previously treated HCC	ORR = 10%; DCR = 61%;	HR = 0.56 (95% CI, 0.42 – 0.76; p <0.001)	HR = 0.89 (95% CI, 0.69 – 1.15; p = 0.33)	Almost a quarter of brivanib patients withdrew due to toxicity	(107)
EGFR	Erlotinib	8xII	342	NA	NA	Meta-analysis for Erolitinib plus Bevacizumab in advanced HCC	Pooled ORR = 12.6%	16-week PFS = 50.2% (95% CI, 38.2 – 62.2)	12-month OS = 44.9% (95% CI, 36.8 – 53.0)	Outcomes data not uniform across included studies	(123)

HCC, Hepatocellular Carcinoma; MET, MET Proto-Oncogene, Receptor Tyrosine Kinase; c-Met, mesenchymal-epithelial transition factor; DCR, Disease Control Rate; ORR, Objective Response Rate; HR, Hazard Ratio; NA, Not applicable/Not reported; PFS, Progression free survival; TGF-β, Transforming Growth Factor-β; AFP, alpha fetoprotein; OS, Overall Survival; ULN, Upper Limit of Normal; TTP, Time To Progression; FGFR, Fibroblast Growth Factor Receptor; EGFR, Epidermal Growth Factor Receptor; NB: Start and end dates are mentioned from articles/abstracts cited. If not available, start and primary completion dates from clinicaltrials.gov have been noted.

**Table 2:** Ongoing/Expected studies with evolutions of previously evaluated molecular targets.

Target	Drug	Mechanism of Action	Study Design (in brief)	Phase	NCT Identifier	Start/Expected Completion	Sample Size (Estimated)	Location
TGF-β	Livmoniplimab	mAb targeting GARP: TGF-β1 complex, blocks active TGF-β release	Livmoniplimab plus Budigalimab (ICI) for previously treated (ICI treatment) HCC	II	NCT05822752	Start: 09/2023 End: 12/2026	120	USA, Europe, Asia
FGFR	Futibatinib	Highly selective, irreversible inhibition of FGFR1–4	Futibatinib plus Pembrolizumab (ICI) for previously treated HCC	II	NCT04828486	Start: 05/2021 End: 05/2025	25	USA
MET/c-Met	ABBV-400	ADC: c-Met–targeting antibody telisotuzumab conjugated to a potent topoisomerase 1 inhibitor (Top1i) payload	Expected soon		Expected soon			

TGF-β, Transforming Growth Factor-β; GARP, Glycoprotein-A Repetitions Predominant; ICI, Immune Checkpoint Inhibitor; HCC, Hepatocellular Carcinoma; FGFR, Fibroblast Growth Factor Receptor; MET, MET Proto-Oncogene, Receptor Tyrosine Kinase; c-Met, mesenchymal-epithelial transition factor; ADC, Antibody-Drug Conjugate.

## Novel targets under investigation

While well-established oncogenic pathways like c-Met/HGF, TGF-β, and FGFR remain important therapeutic targets for the development of novel therapies or combinations in HCC, several new targetable pathways have been identified for HCC in the last few years.

The Wnt signaling pathway serves many vital functions in cell proliferation and differentiation. The pathway can be divided into β-catenin-dependent (also known as canonical) signaling and β-catenin-independent signaling sub-pathways (126). Dysregulation of “canonical” Wnt/β-catenin signaling is commonly seen in HCC (127). There are multiple ways in which this pathway can become aberrantly activated and cause the development and progression of HCC. Numerous studies indicate that mutations in *CTNNB1*, the gene encoding β-catenin, in HCC tumors are linked to a better prognosis and are linked with therapy responsiveness. On the other hand, studies have also suggested that the accumulation of β-catenin in the cytoplasm and nucleus is linked to more poorly differentiated tumors, vascular invasion, and cell proliferation in HCC (126–128). Transducin β-like protein 1 (TBL-1) binds to TBL1 receptor 1 and is known to function as a master regulator of the Wnt signaling pathway by promoting downstream transcription of β-catenin (129, 130). Tegavivint (BC2059) is a novel inhibitor of TBL-1 that prevents the binding of TBL-1 to β-catenin and promotes β-catenin degradation. Anti-tumor activity with the use of tegavivint has been shown in pre-clinical studies involving desmoid tumors, osteosarcoma, adrenocortical carcinoma (ACC), and acute myeloid leukemia (AML) stem/blast progenitor cells (BPCs) (131–134). A phase I dose escalation study (NCT03459469) of tegavivint in desmoid tumors showed adverse events consistent with expected safety outcomes (135). Investigation of the utility of tegavivint in patients with advanced HCC recently commenced in a phase I/phase II exploratory study (NCT05797805). Notably, this study employs a biomarker-selected population, with either AXIN1 or CTNNB1 mutations being required for enrollment, except for patients enrolled in the single-agent dose escalation part of the trial. Patients are required to have received at least one prior line of systemic therapy with a PD-1/PD-L1 inhibitor or have documented intolerance or contraindication to ICI use. The planned phase 2 component of the study involves testing the combination of tegavivint with a PD-1/PDL-1 inhibitor (136). Furthermore, the combination of tegavivint with the histone deacetylase inhibitor panobinostat (LBH589) has shown significant therapeutic effects in myeloma cell lines (137). This may also represent a potential therapeutic combination that can be utilized for patients with HCC.

*MYC*, the gene encoding the c-Myc oncoprotein, is a “master regulator” that controls various aspects of cellular growth regulation and cellular metabolism (138). The *MYC* proto-oncogenes are known to encode transcription factors that are frequently activated oncoproteins in a wide array of human cancers (139, 140). The role of c-Myc in promoting hepatic tumorigenesis has been well described, and high c-Myc expression is associated with a poorer prognosis (141–144). In a study analyzing chemically-induced liver cancer mouse models, c-Myc expression was found to increase with hepatic injury but not in normal liver tissue (145). C-Myc is also known to interact with hypoxia-inducible factor-1α (HIF-1α) in hepatic tumorigenesis. HIF-1α and c-Myc interact to promote the expression of the *VEGFA* gene, which then drives pathological tumor angiogenesis (146, 147). Until recently, due to a lack of a structured binding pocket and its tightly autoregulated expression, c-Myc has been considered “undruggable”. OTX-2002, a first-in-class, programmable mRNA therapeutic that regulates *MYC* gene expression by epigenomic modulation, is currently being evaluated for use in patients with HCC and other advanced solid tumors in the MYCHELANGELO I study (NCT05497453) (148, 149). Eight patients evaluated at initial doses showed evidence of on-target epigenetic changes and decreases in MYC mRNA expression levels (150). Further investigation is ongoing.

Glypican-3 (GPC3), previously called MRX7, is a membrane-associated heparan sulfate proteoglycan that is up-regulated in hepatocellular carcinoma (HCC), especially in poorly-differentiated subsets, with absent or scarce expression in normal liver tissue (151, 152). Targeting GPC3 through novel GPC3-based immunotherapies, such as CAR-T and T cell receptor (TCR) engineering T cell therapy, has generated worldwide attention. The safety of GPC3 directed CAR-T cell therapy in HCC is being evaluated in a phase I first in human dose escalation trial (NCT05003895) (153). The HCC microenvironment also contains an abundance of TGF-β, which may blunt natural or drug-stimulated anti-tumor immunity (74). This finding underlies the rationale for the development of AZD5851, a GPC3 CAR-T “armored” with dominant-negative TGFβRII, which binds to TGFβ but does not result in CAR-T cell inhibition, in a multicenter phase I/II study evaluating the use of AZD5851 in patients with GPC3+ advanced or recurrent hepatocellular carcinoma (NCT06084884). Briefly, study treatment will include three doses of lymphodepleting chemotherapy (fludarabine and cyclophosphamide), followed by one dose of AZD5851 administered by intravenous (IV) infusion (154).

Palmitoyl-protein thioesterase 1 (PPT1) is a lysosomal protein which plays an important role in the intracellular catabolism of lipid-modified proteins. Dysregulated lysosomal activity and mammalian target of rapamycin (mTOR) complex 1 signaling has been shown to play a role in the development of resistance to chemotherapy and targeted therapies in cancer cells (155–157). The role of targeting autophagy has been previously explored by using hydroxychloroquine (HCQ) in other solid tumors, which also acts upon PPT1 to augment its autophagy modulating effects (158, 159). In the context of HCC and its current therapies, preclinical data from murine melanoma models have shown that PPT1 inhibition enhances the anti-tumor activity of immune checkpoint inhibition using an anti-PD-1 antibody (160). The potential utility of PPT1 blockade in conjunction with ICIs is being evaluated in an ongoing phase IIb study using GNS561/ezurpimtrostat, a novel inhibitor of PPT1, which has shown promising anti-tumor activity in pre-clinical models (NCT05448677) (161, 162). After a safety lead in phase, patients will be randomized to receive ezurpimtrostat in addition to the combination of atezolizumab and bevacizumab, compared to the control arm of atezolizumab and bevacizumab alone (163).

**Table 3** provides an overview of ongoing investigations for some novel targets.

**Table 3:** Ongoing studies with notable novel molecular targets.

Target	Drug	Mechanism of Action	Study Design (in brief)	Phase	NCT Identifier	Start/Expected Completion	Sample Size (Estimated)	Location
Wnt/β-catenin	Tegavivint	TBL-1 inhibiton: that prevents the binding of TBL-1 to β-catenin and promotes β-catenin degradation	Tegavivint dose escalation followed by Tegavivint plus Pembrolizumab (ICI) in previously treated HCC; *CTNNB1* or *AXIN1* mutations required in part 2	I/II	NCT05797805	Start: 09/2023 End: 05/2025	108	USA, Canada
MYC/c-Myc	OTX-2002	Programmable RNA causing epigenomic modulation of c-Myc expression	Part 1: OTX-2002 dose escalation in advanced solid tumors Part 2a: OTX-2002 + TKI One in previously treated HCC Part 2b: OTX-2002 + TKI Two in previously treated HCC Part 2c: OTX-2002 + ICI in previously treated HCC	I/II	NCT05497453	Start: 08/2022 End: 06/2025	190	USA, Asia
GPC3	AZD5851	GPC3 CAR-T armored with dominant-negative TGFβRII	Lymphodepleting Chemotherapy followed by AZD5851 in previously treated HCC	I/II	NCT06084884	Start: 11/2023 End: 12/2027	84	USA, Asia
PPT1	Ezurpimtrostat	PPT1 inhibition which regulates autophagy	Ezurpimtrostat + Atezolizumab/Bevacizumab vs. Atezolizumab/Bevacizumab alone for treatment-naïve HCC	II	NCT05448677	Start: 12/2022 End: 03/2024	3	France

TBL-1, Transducin β-like protein 1; ICI, Immune Checkpoint Inhibitor; HCC, Hepatocellular Carcinoma; RNA, Ribonucleic Acid; GPC3, Glypican-3; PPT1, Palmitoyl-protein thioesterase 1; NB: Start and primary completion dates from clinicaltrials.gov have been noted.

## Discussion

Systemic therapy for HCC is rapidly evolving, yet there remains a significant unmet need to develop novel targeted therapies and biomarkers. Although many targeted therapy trials in HCC have failed to meet their primary endpoints or complete accrual, they have still provided valuable insights to inform the design of the next generation of clinical trials.

The development of c-Met inhibitors exemplifies this idea. Studies investigating tepotinib, capmatinib, golvatinib, and tivantinib were heterogeneous with regards to the inclusion of c-Met overexpression as part of the eligibility criteria, and there was no uniformly defined threshold for c-Met overexpression (46, 48–50, 164). Improved biomarker selection might lead to the identification of therapeutically relevant patient subsets in future trials. A high rate of screening failures was also noted across many of these studies due to multiple factors. Current studies exploring c-Met as a therapeutic target commonly employ biomarker-based selection and combination therapies or co-targeting of additional pathways (57). Promising results with cabozantinib indicate that the inhibition of the VEGF pathway may synergistically contribute to anti-tumor activity for HCC patients in whom the c-Met pathway is being targeted. More stringent selection based on c-Met status may also result in more promising results with c-Met inhibition in HCC. Combinations of c-Met inhibitors with immune checkpoint inhibitors are yet to be explored in larger studies. Data from the combination of emibetuzumab with ramucirumab suggest that patient selection for trials involving combinations with c-Met inhibitors based on c-Met expression holds great promise (54).

Similarly, although prior trials with the agents targeting TGF-β did not lead to any drugs approved for HCC treatment, the potential utility of this pathway remains significant. The activity of TGF-β as a regulator of immune responses is being leveraged in its currently ongoing combination trials with ICIs. Further, the combined inhibition of the TGF-β and VEGF pathways is another important therapeutic avenue for future studies (64, 94, 97). Inhibition of the Wnt/β-catenin signaling pathway also represents an exciting therapeutic avenue for HCC, with promising results from preclinical studies. Co-targeting strategies involving additional oncogenic pathways have also been developed on the basis of additional preclinical studies. For example, co-inhibition of the RAS/RAF/MAPK pathway and co-inhibition of cyclin dependent Kinases 4/6 have both shown promising results and represent potential combination therapies for future trials (165–167). Indeed, the potential of targeting the c-Myc pathway, traditionally considered “undruggable”, holds tremendous clinical promise, as does the potential addition of epigenomic modulators as a drug class in the targeted therapy armamentarium (168). GPC3 is a novel target that is highly specific in its expression in HCC tissue compared to its absence in normal tissues (151, 152). Hence, it represents an exciting novel target for HCC, and investigations using novel strategies like CAR-T cell therapy which target GPC3 are ongoing. Similarly, the modulation of cancer cell lysosomal activity by blocking PPT1 represents another promising new target, especially in the context of the enhancement of ICI activity with PPT1 blockade (160).

There is an increasingly recognized need for biomarker-driven patient selection for studies evaluating systemic therapy regimens in HCC. In the era of precision medicine, with the rapid increase in the use of next generation sequencing (NGS), significant improvements in patient outcomes have been enabled with the use of biomarker-based targeted therapeutic regimens in several solid tumors such as lung cancer, melanoma, and intrahepatic cholangiocarcinoma (169–172). Results from the studies involving c-Met targeting agents point towards the utility of biomarker-driven patient selection in HCC (54). Several ongoing studies employ a biomarker-based selection strategy. For example, in the phase I/II study evaluating tegavivint, mutations in either *CTNNB1* or *AXIN1* genes will be required for enrollment in the arm evaluating the combination of tegavivint with pembrolizumab in the planned phase 2 component of the study (136). Similarly, FGF19 expression in tumor cells is required for enrollment in the ongoing study evaluating futibatinib (117).

With the increasing utilization of NGS based tissue assays in routine clinical practice, there is growing interest in genome-directed therapies for HCC patients. Tumor genomic profiling has the potential to identify patients who are most likely to respond to certain systemic therapies and those who develop resistance to such therapies. A prospective analysis of tumor DNA from 127 patients, as reported by Harding et al., showed that alterations in the PI3K-mTOR pathway were associated with lower disease control rates, shorter mPFS (HR, 3.8; 95% CI, 2.0 – 7.5; p <0.0001), and shorter mOS (HR, 10.4; 95% CI, 1.21 – 5.31; p = 0.01). WNT-activated tumors were found to have a shorter mPFS with ICI (HR, 9.2; 95% CI, 2.9–28.8; p < 0.0001) than non-altered tumors. 24% patients were reported to have at least one actionable mutation, namely in the *TSC1/2*, *PTEN*, *FGF19*, *MET*, *IDH1*, *HRAS*, *NRAS*, and *PI3KCA* genes (173). Limousin et al. recently reported the results from a tertiary care center in France evaluating the use of molecular-based targeted therapies in HCC and hepato-cholangiocarcinoma (H-CCK) patients refractory to atezolizumab/bevacizumab. The pilot study results indicate a reasonable overall feasibility of this approach. Briefly, among 14 patients with actionable genomic alterations, nine were given an adapted targeted therapy. Three patients (two with H-CCK and one with HCC) having alterations in CDK4, HER2, and TSC2 achieved disease control with palbociclib, trastuzumab/olaparib, and everolimus, respectively. However, the other six HCC patients had disease progression despite various genomically-guided treatment attempts (174). Liquid biopsy assays may also represent a feasible tool for the detection of actionable or predictive genomic alterations in HCC patients and reveal potential biomarkers for the monitoring of targeted therapies (175, 176).

Perhaps the greatest area of unmet need in HCC systemic therapy remains for patients with moderate liver dysfunction (CP class B) (177). Limited data focusing on this population points towards the potential to improve patient outcomes with systemic therapies for these patients (32, 33). The biological plausibility of the efficacy of drugs like c-Met inhibitors in this patient population makes trial design a pragmatic and potentially feasible approach to consider for this patient population (36, 42).

## Conclusions

With the advent of immune checkpoint inhibitors and targeted therapies, the systemic therapy landscape for HCC has undergone a paradigm shift in the past few years. Although several clinical trials involving targeted therapies for HCC have not yielded tangible clinical benefit, these therapeutic targets, including c-Met, TGF-β, and FGFR, remain relevant to current and future investigations involving novel agents and modified patient selection and study design. The Wnt/β-catenin signaling pathway, c-Myc, GPC3, and PPT1 represent exciting novel targets for HCC. CAR-T cell therapy and antibody-drug conjugates are additional novel therapy modalities for HCCs under current investigation. Genomic biomarker-based patient selection for targeted therapies holds great promise and informs the design of the next generation of clinical trials investigating targeted therapies for HCC.
